# The Association Between Psychological Distress and Young People Engaging in Family Violence: A Data-Linkage Study

**DOI:** 10.1177/08862605261444015

**Published:** 2026-05-28

**Authors:** Maddison Riachi, Benjamin Spivak, Troy McEwan, Nina Papalia

**Affiliations:** 1 Swinburne University of Technology – Centre for Forensic Behavioural Science & Victorian Institute of Forensic Mental Health, Hawthorn, VIC, Australia; 2 University of Kent, Canterbury, United Kingdom

**Keywords:** intimate partner violence/abuse, mental health and violence, youth violence, teen dating violence, dating violence, child to parent violence

## Abstract

Young people engaging in family violence (FV) have higher rates of mental health symptoms and diagnoses than other young people. However, the presence of a time–order relationship between mental ill-health/psychological distress and a young person’s use of violence towards family members is yet to be determined. This study had two aims: the first, to investigate the mental health of young people engaging in FV with consideration to possible differences based on FV characteristics; and the second, to determine whether psychological distress (as indicated by mental health service presentations) occurs contemporaneously with young people’s use of violence towards family members. This Australian study used a pseudo-prospective data-linkage design. The sample consisted of 361 young people recorded by Victoria Police between September 2016 and June 2017 for using FV. This sample of young people was linked to health records from multiple national and state datasets containing data on lifetime public mental health service presentations and diagnoses, as well as publicly subsidised private mental health service presentations from 2010 to 2018. There was a high prevalence of mental health service presentations (74%) in this sample, with 31.3% having a lifetime mental health diagnosis recorded in the public mental health system. Young people using violence across multiple intimate partner or familial relationships had higher rates of psychological distress; however, the same was not found for young people with a police-recorded FV victimisation history. There was also evidence to suggest a temporal relationship between youth family violence (YFV) incidents and psychological distress, with the odds of a young person presenting to a mental health service being higher prior to or around the same time as their police-reported YFV incident. These findings move the evidence base closer to determining a causal relationship between mental ill-health and YFV.

## Introduction

Youth family violence (YFV) is defined as physical (e.g., physical abuse, sexual abuse) or non-physical (e.g., coercive control, psychological abuse, financial abuse) abusive behaviour used by a young person aged under 25 years towards a family member (e.g., parent or sibling), or current or former intimate partner – regardless of cohabitation (Sheed, McEwan, Papalia et al., 2023). The inclusion of young people up to 25 years old reflects the increasing recognition of delayed brain maturation for individuals into their twenties (Cohen et al., 2016; Scott et al., 2016) and that many services for youth provide support to young people up until the age of 25 years (McGorry et al., 2022). Young people engaging in FV behaviours have higher rates of mental health conditions and report more mental health symptoms than other young people (Contreras & Cano, 2014; Kennedy et al., 2010). However, it is unclear whether mental health conditions and associated psychological distress occur contemporaneously with young people’s use of violence towards family members. The current study aims to address this gap in the literature whilst investigating mental health service presentations present in this cohort.

YFV is attracting increasing attention as a behaviour of concern in Australia and internationally. Twenty percent (*n* = 17,675) of Australians proceeded against by police for FV-related offences in 2022–2023 were aged between 10 and 24 years old (Australian Bureau of Statistics, 2024), with 24%–35% having further police contact for FV within 6 months (Sheed, McEwan, Papalia et al., 2023). Mental ill-health is a common individual-level factor across all YFV sub-types (Calvete et al., 2013; Thomas et al., 2019; Tucker et al., 2013); however, there is little understanding of how mental ill-health may be related to YFV use. If psychological distress or mental ill-health is shown to increase the likelihood of subsequent YFV behaviours, this could provide a potential avenue for targeted mental health-related interventions to reduce YFV. These interventions may also hold long-term benefits for young people engaged in YFV, as research indicates that untreated mental health conditions in youth are associated with adverse long-term outcomes, including educational disruption, employment difficulties, relationship instability, and increased risk of ongoing violence perpetration and victimisation across the lifespan (Asselmann et al., 2018).

### Mental Health of Young People Engaging in Family Violence

Mood disorders, disruptive, impulse-control and conduct disorders, and neurodevelopmental disorders have been shown to be prevalent in samples of young people who use FV (Calvete et al., 2013; Dardis et al., 2015; Foshee et al., 2001; Mathis & Mueller, 2015; Phillips et al., 2018; Tucker et al., 2013). The severity of psychological distress in this population is evidenced by a custodial sample of 100 child-to-parent abuse (CPA) users (a sub-type of YFV) in Florida reporting substantially higher rates of psychiatric admissions (20%), psychiatric medications (29%), and suicide attempts (19%) compared to those that had not engaged in this behaviour (7.2%, 13.6%, 3.6%, respectively; Kennedy et al., 2010). More recently, Papalia et al. (2024) found that young females (aged 0–17 and 18–24) and young males (aged 18–24) with police contact for YFV were significantly more likely to present to emergency services for suicide or self-harm than young people in the general population.

The relationship between mental health and YFV use appears to be influenced by experiences of victimisation. According to Peck et al. (2022), adolescents with a mental health diagnosis had nearly double the rate of child-to-parent violence reoffending compared to those without – but only when there was no police-recorded history of exposure to parental intimate partner violence in childhood. The complex role of victimisation is further demonstrated by research showing that individuals who have been both victims and users of YFV have elevated mental health risks. For example, Dantchev et al.’s (2019) longitudinal study of 3,881 young people aged 12 to 24 years in the United Kingdom found that young people who had both abused and been abused by their sibling had higher odds of a clinical diagnosis of depression at age 24 years (*OR* = 1.91, 95% CI [1.33, 2.72]) compared to those in single roles or with no involvement. Similarly, Thomas et al. (2019) reported that young women who had both engaged in youth intimate partner abuse (YIPA; a sub-type of YFV) and been victim-survivors of YIPA had significantly higher odds of depression (*OR* = 3.52 [2.43, 5.09]) and anxiety (*OR* = 4.98 [3.29, 7.55]) compared to sole victim-survivors, sole users of abuse, and those with no YIPA involvement.

Whilst these findings highlight the complex mental health needs of young people engaging in YFV, researchers emphasise the potentially tautological nature of mental ill-health within YFV populations. For example, a young person’s use of violence could be viewed as a disruptive pattern of behaviour which is required to meet diagnostic criteria for some mental health disorders (Simmons et al., 2018). Others have also highlighted the importance of considering a young person’s victimisation history, as child abuse history has been found to mediate the relationship between poor mental health and YFV use (Ralph et al., 2025). However, Ralph et al.’s (2025) recent secondary analysis of data from the Adolescent Family Violence in Australia Study (Fitz-Gibbon et al., 2022) – a national online survey of 5,021 young people aged 16 to 20 years old – suggested that poor mental health still held an independent direct effect on YFV use, although the type of mental health condition/symptoms and timing of such was not specified.

Furthermore, consideration needs to be given to the potential two-way nature of the relationship between mental health and YFV. Although no published studies have investigated this issue within a YFV sample, the adult intimate partner violence literature provides evidence for a two-way relationship, with studies showing that perpetration of IPV is associated with poor mental health outcomes, and conversely, poor mental health is associated with IPV perpetration (Chatterji & Heise, 2021; Spencer et al., 2019). These findings highlight the complexity of the mental health–violence relationship and underscore the importance of examining the temporal sequencing of these phenomena to better understand potential causal pathways.

### The Relationship Between Mental Ill-Health, Psychological Distress, and Incidents of YFV

While the abovementioned studies provide important insights into the association between mental health concerns and young people’s use of FV, they do little to elucidate the nature of the relationship between these two phenomena. Whether they are temporally associated, and any potential time–order is unclear. The experience of mental ill-health is dynamic, therefore changes over time. Thus, the presence of a mental health disorder at one time point does not necessarily mean the presence of active symptoms, or of distress associated with symptoms, at another time point. To date, no studies investigating the mental health of young people engaging in FV have explored the time–order relationship between mental ill-health and a young person’s use of FV. This has been investigated in the adult general violence literature (Skeem et al., 2016), with the conclusion that active symptoms of mental illness may immediately precede incidents of violence for some adults, but for the large majority, symptoms of illness are not the primary precipitating factor for violence (Skeem et al., 2016). The authors concluded that other factors, such as anger or social deviance, may also be key in reducing an individual’s use of violence (Monahan & Steadman, 2012). By contrast, Spivak et al. (under review) investigated this relationship in the context of adult FV using a broader measure of mental health distress (i.e., contact with mental health services). They found that contact with public mental health services was temporally associated with police reports of FV within the same quarter, suggesting a more proximal relationship between acute mental health service use and FV incidents. These differing findings underscore the importance of examining the nature and timing of mental health difficulties in relation to FV, particularly in youth populations where this has not yet been explored.

Although no published studies have directly investigated the time–order relationship between mental health and YFV use, there is some indirect evidence for a temporal relationship from qualitative studies. For instance, Sheed, Maharaj et al. (2023) used qualitative methods to review police narratives of incidents of CPA in Australia involving young people aged between 10 and 24 years old and found poor mental health to be one of the commonly identified situational antecedents. Ralph et al. (2025) found similar results in a survey of 5,021 Australian young people (aged 16–20). Twenty percent of the sample identified as having used violence against a family member, with qualitative analyses indicating that young people identified mental health symptoms as a key factor in their use of FV. These findings are consistent with international qualitative research, where victim-survivors of YFV report a young person’s violence to be, in part, due to psychological distress around the time of FV incidents (Williams et al., 2017). Whilst these studies have provided a valuable foundation to support the hypothesis that psychological distress occurs contemporaneously with young people engaging in violence towards family members, further research utilising larger sample sizes, objective and timely measures of psychological distress, and longitudinal study designs are needed to make more robust inferences regarding the time–order relationship between a young person’s psychological distress and their use of violence towards family members.

### The Current Study

A pseudo-prospective data-linkage design involving a cohort of police-recorded YFV users was used to investigate two aims and address some limitations of existing research. The first aim was to investigate the mental health of young people engaging in FV, with consideration given to the potential impact of FV characteristics. The second aim was to determine whether psychological distress (conceptualised more broadly than self-harm), as indicated by presentations to mental health services, occurs contemporaneously with young people’s use of violence towards family members.

These aims were operationalised through the following research questions:

1) What is the prevalence of mental health service presentations and associated mental health diagnoses of young people engaging in family violence?
2) Do the mental health service presentations and associated mental health diagnoses of young people engaging in family violence vary according to different characteristics of family violence (i.e., use of violence across multiple family relationships, history of family violence victimisation)?
3) Is there a time–order relationship between YFV incidents and mental ill-health or psychological distress (as indicated by mental health service presentations)?

It was hypothesised that mental health disorders recorded within a cohort of young people using YFV would be similar to those that have been identified in previous research (i.e., mood disorders, disruptive, impulse-control and conduct disorders, and neurodevelopmental disorders). However, the investigation of mental ill-health and psychological distress in this cohort through presentations to mental health services was more exploratory, given the lack of prior research. It was hypothesised that young people who have a history of FV victimisation would have higher rates of mental health disorders and mental health service presentations; however, the same could not be hypothesised for those who use violence across multiple relationships due to a lack of literature to guide such a hypothesis. Finally, it was hypothesised that mental ill-health or psychological distress (evidenced through mental health service presentations) would precede incidents of young people using violence in intimate or familial relationships.

## Method

The study adopted a pseudo-prospective design using individual record linkage across population-level administrative police and mental health-related datasets.

### Data Sources

#### Family Violence Sample and Characteristics

The sample was derived from a wider project examining how changes to police FV risk assessment and management practices affected FV recidivism and well-being of both victims and perpetrators in the Australian state of Victoria (McEwan et al., 2019; Spivak et al., 2021). The sample used in the current study was drawn from the control sample in the broader project, which did not receive any non-standard policing practice in response to reports of FV. Due to reliance on an existing sample of individuals with police involvement for FV, no matched comparison group without police-recorded FV was available, which is a limitation of the present analysis.

The control sample from which this study’s sample was drawn consisted of 1,588 randomly selected FV incidents recorded by Victoria Police between 1 September 2016 and 30 June 2017. Random selection of these incidents was conducted by Victoria Police and not the research team. These incidents were selected from a single police division in the northern area of Melbourne, Australia. This division neighboured two divisions in which the intervention was implemented in the broader study. Population sociodemographic characteristics of the division are presented in the Supplemental Information (Table S1). The population is comparable to the Victorian population on sex, education level, and median income, but includes more younger people (64.5% <45 years vs. 60.2%), fewer Indigenous Australians (0.57% vs. 0.80%), and fewer individuals born in Australia (59.8% vs. 64.1%), compared with the Victorian population.

Victoria Police provide all policing services across Victoria. Officers are required to record an FV incident whenever they judge FV to be present, regardless of whether it constitutes a criminal offence or results in a charge. FV is defined by the Family Violence Protection Act (2008) as:

Behaviour by a person towards a family member of that person if that behaviour is physically or sexually abusive; or is emotionally or psychologically abusive; or is economically abusive; or is threatening; or coercive; or in any other way controls or dominates the family member and causes that family member to fear for the safety and wellbeing of that family member or another person. (Family Violence Protection Act, 2008, s. 5).

FV incidents to which police respond are recorded in the Law Enforcement Assistance Programme (LEAP) database. Each incident has an identified Respondent (i.e., person alleged to have perpetrated FV) and affected family member (AFM; i.e. primary victim). Full police records of FV and any FV incidents in the 7 months after 30 June 2017 were extracted from LEAP for every individual in the control sample as part of the broader study’s methodology. This meant that the full scope of police contact for FV was available, from the date of their first recorded FV incident (which varied for each individual) until the end of January 2018. For the ease of the reader, young people who have used violence in an intimate or familial relationship will be referred to as Respondents throughout the method and results to accurately reflect the data, while the Discussion will revert to discussing young people who have used YFV for ease of comparison to other literature.

The sample used in the study consisted of any individual in the control sample who had an FV incident in which they were involved as a Respondent while aged below 25. The first incident within the sampling period (1 September 2016 and 30 June 2017) where the young person was listed as a Respondent is referred to as the ‘index incident’ in this paper.^1^ For each index incident, demographic information (i.e., sex and age – which were controlled for in all analyses), and the relationship between the Respondent and AFM was provided. It is noted that for research question 3, all FV incidents committed by the Respondents in this study’s sample between January 2010 and the end of January 2018 were used as the independent variable. Although lifetime FV histories were available, this timeframe was selected for the temporal analyses to align with the mental health contact dates available (details provided in the next section).

Variables extracted from LEAP and used in this study were as follows: Respondent age at index incident (under 18/18 and above); sex (female/male); relationship with the victim and any previous lifetime police-recorded FV victimisation (yes/no). Additional demographics, such as cultural or gender identity, were not available as they were not collected by the police at the time. It is noted that these were also not routinely captured in the administrative health data used (details below), and the lack of diversity indicators is a limitation of the study. Victim demographics (i.e., age and sex at index incident) were also extracted from LEAP but were not used in this study. An additional variable was created from LEAP lifetime pre-index incident records to capture patterns of police-reported YFV use. The ‘violence use in multiple relationships’ variable indicated whether the young person had been a Respondent in multiple FV incidents involving different AFMs (e.g., two incidents that, respectively, record their mother and their father as an AFM would result in a ‘yes’ code).

#### Mental Health and Psychological Distress

The current study used contacts with mental health services as a proxy for mental ill-health or psychological distress, given that individuals typically present to mental health services when they are experiencing a level of psychological distress that warrants professional care.

##### Private Community Mental Health Service Presentations

Data on each young person’s contact with mental health providers outside of public mental health services (e.g., privately practicing health practitioners) were extracted from the Medicare Benefits Scheme (MBS). The MBS is a part of Australia’s nationalised health care system and provides free or subsidised physical and mental health services. The MBS database holds records of the number and types of eligible services accessed by individuals across all Australian states and territories, though diagnostic information is not recorded. The MBS records used in this study dated from January 2010 to January 2018, with the earlier date chosen due to funding limitations for data extraction for the broader project. Using MBS item numbers and descriptions, mental health service presentations were defined as relating to general practitioners, psychiatrists, psychologists, or allied health practitioners (i.e., clinical social workers). Individuals were assigned a numerical value based on the number of contacts – for example, an individual with six contacts with a psychologist and two contacts with a psychiatrist between 2010 and 2018 would be assigned a count of eight.

##### Public Mental Health Service Presentations and Diagnoses

Victorian public mental health services data are recorded in the Client Management Interface/Operational Data Store (CMI/ODS), held by the Victorian Agency for Health Information. The Victorian public mental health system provides specialist mental health care to those who are seriously affected by mental illness and cannot receive adequate care through a primary care provider alone. It includes in-patient, out-patient, and emergency room mental health services. Public mental health service records used in this study spanned between January 2010 and January 2018 for service contacts (to accord with available MBS service contacts data), but mental health diagnoses were extracted across the lifetime until January 2018, as mental health diagnoses are not always provided at each contact with a public mental health service.

Data extracted included the type and date of service contact, services received, and mental health disorder diagnosis/diagnoses as classified using the International Classification of Diseases 11th Edition (ICD-11). Service contacts were categorised as either community public mental health service presentations or emergency/in-patient/acute public mental health service presentations (the same coding approach as adopted by Spivak et al. [under review]). Specific details related to the reason for contact were not available (e.g., suicidal ideation or self-harm); however, public mental health services in Victoria typically manage the more complex and acute mental health presentations that include behaviours such as suicide and self-harm (Short et al., 2010). Like the MBS data, individuals were assigned a numerical value for each category based on the number of contacts.

Diagnoses were separated into the following categories and coded as present or absent: anxiety and fear-based disorders; mood disorders; neurodevelopmental/childhood disorders; personality disorders; schizophrenia/psychotic disorders; stress disorders; substance use disorders (see Table S2 for coding scheme). A separate variable capturing the presence of any diagnosis (yes/no) was also created.

##### Overall Mental Health Service Presentations

An overall mental health service contact variable was created using both the MBS and public mental health service data. Mental health service presentations were coded into three tiers, reflecting increasing acuity. Private mental health contacts were classified as tier one, community public mental health contacts classified as tier two, and emergency/in-patient/acute public mental health contacts were classified as tier three. Counts across all three tiers were combined to capture the total number of contacts with any mental health service.

### Data Linkage

Linkage of the FV sample to health datasets was conducted by data analysts at Australian Institute of Health and Welfare and the Centre for Victorian Data Linkage. Identifying information (e.g., full names, gender, and date of birth) from the sample was used by data analysts to find probabilistic linkages between the sample and MBS and Victorian public mental health services data. Data from both health datasets were then extracted and merged with demographic and FV data drawn from police records. All identifying information was removed at this stage before the researchers accessed it. Given the number of data sources, Table S3 in the Supplemental Information summarises the sample and outcome data sources, timeframes, and their relationship to each research question.

### Ethics Approval

This study was approved by three independent human research ethics committees; Swinburne Human Research Ethics Committee, the Victoria Police Human Research Ethics Committee Human Research Ethics Committee, and the Australian Institute of Health and Welfare (AIHW) Research Ethics Committee. Reciprocal approval was granted by the Victorian Department of Health based on AIHW ethics approval. All methods were carried out in accordance with the relevant guidelines and regulations.

### Data Analysis

Data analysis was conducted using the statistical programming package RStudio Version 4.0.3 (Team, 2020) with packages readxl (Wickham & Bryan, 2019), psych (Revelle, 2021), ggplot2 (Wickham, 2016), dplyr (Wickham et al., 2021), lme4 (Bates et al., 2015), and MASS (Venables & Ripley, 2002). Descriptive statistics were used to characterise the sample of Respondents, including to determine the prevalence and frequency of mental health service presentations, and the prevalence of mental health disorders (research question one).

To address research question two, negative binomial regression was used to determine the association between violence characteristics (i.e., FV victimisation history and FV use towards multiple family members) and the number of mental health service presentations, controlling for Respondent sex and age at index incident. Logistic regression was conducted with the same variables to determine the association between violence characteristics and the odds of receiving any mental health diagnosis.

Analyses to determine the time – order of YFV incidents and psychological distress (as indicated by mental health service presentations) were planned (research question three); however, they were not feasible given the low sample size. Instead, three random coefficients multilevel logistic regression models were calculated to determine the probability of a mental health service contact occurring in any given three-month period (quarter of interest) if there was a YFV incident in the quarter before, in the same quarter, or in the quarter immediately following the quarter of interest. This alternative analysis still provides an indication of temporality in the association between YFV and psychological distress. In each model, Respondent sex, Respondent age at time of index incident, FV victimisation history, and the use of violence in multiple relationships were treated as time invariant. YFV incidents and mental health service presentations between January 2010 and January 2018 were used in these models. Further details of these analyses have been provided in the Supplemental Information (Figure S1).

## Results

### Sample Characteristics

There were 361 Respondents in the sample. The mean age of individuals at the time of their index incident was 20.36 years old (*SD* = 3.45), and the majority of the sample was male (*n* = 270, 75%), consistent with previous research that consistently identifies a predominance of males in samples with police contact for YFV (Simmons et al., 2018). Most youth were aged between 18 and 24 years (*n* = 232; 64%). One hundred and thirty-five (37%) individuals had prior police-recorded FV victimisation, and 187 (52%) were recorded as having engaged in violence in multiple relationships.

### Prevalence and Frequency of Mental Health Service Presentations and Prevalence of Mental Health Diagnoses (RQ 1)

Seventy-four percent (*n* = 268) of Respondents had contact with a mental health service between 2010 and 2018. Over half (59%; *n* = 212) had at least one contact with a Tier One service, 32% (*n* = 115) had at least one contact or admission with a Tier Two service, and 26% (*n* = 94) had at least one contact or admission with a Tier Three service. Standard descriptive statistics did not provide sufficient information about the nature of mental health service use due to the distribution of the data being negatively skewed. Therefore, a five-point summary (minimum value, first quartile, median, third quartile, and maximum value) of mental health service contacts/admissions for Respondents who presented to mental health services at least once between January 2010 and January 2018 is provided in Table 1. The median number of mental health service contacts across January 2010 to January 2018 for the entire sample was 14, with the minimum number of contacts being 1 and the maximum being 2,553. Sex- and age-specific descriptives are detailed in Table 1 (see Table S4 for these descriptive statistics by mental health service tier).

**Table 1.** Descriptive Statistics for Frequency of Mental Health Service Presentations and Prevalence of Mental Health Diagnoses Among Respondents (*N* = 361).

		Total	Sex		Age	
Variable	Statistic		Male	Female	Under 18	18–24
Mental health service presentations^a^	*n* ^c^	21,043	13,160	7,883	4,162	16,881
	Min	1	1	1	1	1
	Q1	5	4	6	3	6
	Med	14	13	31	14	15
	Q3	63	54	110.5	60.5	63
	Max	2,553	2,553	1,558	689	2,553
Any diagnosis^b^	*n* ^c^	113	80	33	21	92
	%	31.3	30	36	28	32

a Mental health service presentations relate to those with at least one service contact between January 2010 and January 2018.

b The mental health diagnoses are only recorded for the subset of Respondents with a lifetime public mental health record.

c *n* for mental health service presentations refers to total service contacts in the sample, whereas *n* for any diagnosis relates to the number of individuals with a diagnosis.

*Note*. Due to the negatively skewed distribution of the data, a five-number summary (minimum, first quartile, median, third quartile, and maximum) is presented for mental health service contacts/admissions among respondents who accessed mental health services at least once between January 2010 and January 2018.

Almost one-third (31%) of Respondents had a lifetime mental health diagnosis recorded in public mental health service records (Table 1). The most prevalent diagnoses were substance use (18%; *n* = 65) and stress disorders (17%; *n* = 61), followed by mood disorders (12%; *n* = 43), personality disorders (10%; *n* = 36), neurodevelopmental/childhood disorders (10%; *n* = 36), and psychotic disorders (9%; *n* = 31). Anxiety and fear-based disorders (7%; *n* = 24) had the lowest prevalence among Respondents.

### Association Between Family Violence Characteristics and Mental Health Service Presentations and Mental Health Diagnoses (RQ 2)

#### Mental Health Service Presentations

Controlling for Respondent sex and age at index incident, the negative binomial regression model indicated that Respondents using violence in multiple relationships presented to mental health services at a rate 2.63 times higher than those who were violent in a single relationship (Incident Rate Ratio [IRR] = 2.63, 95% Confidence Interval [CI] [1.64, 4.20]). However, the association between FV victimisation history and mental health service presentations was not statistically significant (IRR = 1.00 [0.62, 1.63], *p* = .994).

#### Mental Health Diagnoses

Controlling for Respondent sex and age at index incident, the logistic regression model determined that the odds of a mental health disorder diagnosis were 1.11 times greater (*OR* = 1.11, 95% CI [1.01, 1.23]) among Respondents who used violence in multiple relationships compared to those who were violent in a single relationship, whereas the association between FV victimisation history and mental health disorder was non-significant (*OR* = 1.07 [0.96, 1.20]).

### Temporal Association Between Mental Health Service Presentations and YFV Use (RQ 3)

The results of the three random coefficients multilevel logistic regression models suggest that there was a temporal association between YFV use (i.e., a YFV incident as Respondent) and contact/admission with any mental health service (Table 2). Specifically, the odds of a mental health service presentation were 1.82 times higher in the quarter (i.e., 3 months) before a YFV incident (*OR* = 1.82, 95% CI [1.03, 2.01]) or in the same quarter as the YFV incident (*OR* = 1.82 [1.20, 2.78]). There was no significant relationship between YFV incidents and mental health service presentations in the following quarter. These analyses controlled for Respondent sex, age at index incident, FV victimisation history, and violence use in multiple relationships.

**Table 2.** Association Between Mental Health Service Presentations and YFV Use Between January 2010 and January 2018.

Time of Mental Health Presentation	Variable	Odds ratio	CI	*p*
Quarter before	YFV incident	1.82	1.03, 2.01	.035
	Sex	0.94	0.58, 1.56	.851
	Age	0.97	0.91, 1.03	.374
	Victimisation history	0.81	0.72, 1.55	.780
	Multiple relationship	1.09	0.71, 1.55	.801
Same quarter	YFV incident	1.82	1.20, 2.78	.005
	Sex	0.94	0.58, 1.54	.807
	Age	0.97	0.91, 1.03	.329
	Victimisation history	0.81	0.55, 1.19	.282
	Multiple relationship	1.09	0.75, 1.60	.651
Quarter after	YFV incident	1.04	0.71, 1.50	.857
	Sex	0.95	0.58, 1.55	.838
	Age	0.98	0.92, 1.04	.428
	Victimisation history	0.67	0.46, 1.04	.080
	Multiple relationship	1.07	0.71, 1.60	.763

*Note.* Reference groups were as follows: violence in multiple relationship = no, victimisation history = no, sex = female. YFV = youth family violence.

## Discussion

This study had two aims: the first, to investigate the mental health of young people engaging in FV behaviours with consideration to possible differences based on FV characteristics; and the second, to determine whether mental ill-health or psychological distress (as indicated by mental health service presentations) occurs contemporaneously with young people’s use of violence towards family members.

### Psychological Distress and Mental Ill-Health Among Young People Engaging in Family Violence

Consistent with the hypotheses, young people engaging in YFV exhibited elevated rates of mental ill-health or psychological distress as evidenced by mental health service presentations and associated mental health diagnoses. Seventy-four percent of respondents had accessed mental health services over the 8-year study period, with a median of 14 contacts with mental health services. Although age-comparable rates of the general population’s contact with public mental health services for the same time period are not publicly available, data on the number of contacts with publicly subsidised mental health providers (i.e., tier one services) indicate that the general Australian population had an average of five contacts between 2010 and 2018 (AIHW, 2021). This suggests that young people engaging in YFV may experience more mental ill-health/psychological distress requiring assistance than the general population.

Nearly one-third of the YFV sample had a recorded lifetime mental health diagnosis associated with public mental health service use (i.e., tier two and three services). Stress disorders (e.g., posttraumatic stress disorders, adjustment disorders; 17%) and mood disorders (e.g., major depressive disorder, bipolar disorder; 12%) were common in this sample, which is consistent with previous research (Calvete et al., 2013; Nowakowski-Sims & Rowe, 2017).

There was also a relatively high incidence of substance use disorders (18%), which had not been identified in previous studies, though it is possible that this is due to methodological differences between this and past studies, including the fact that substance use disorders have not been consistently included when investigating mental health diagnoses. The high incidence of substance use disorders is consistent with research that has identified substance use as a risk marker for YFV use across sex and ethnicity in community, clinical, and custodial samples (Beckmann et al., 2021; Choi et al., 2020), which suggests that future research needs to investigate the time-based relationship between substance use and engagement in YFV.

The relatively high rates of psychotic disorders within the sample are also of note. Approximately 9% of the sample had received a lifetime diagnosis of a psychotic disorder; three times higher than the rate of diagnosed psychotic illnesses among young people (24 and under) as specified by a United Kingdom population-based cohort study, which estimated rates to be 2.8% (Sullivan et al., 2020). Noting that prevalence figures for young people in Australia with psychotic illnesses are not available. This association is somewhat expected given the literature demonstrating the association between psychotic disorders and violence, noting that such findings have predominantly been investigated in adult populations (Fazel et al., 2018), as well as findings from Short et al. (2013), who found that within a community sample of adults with psychotic disorders (as indicated by CMI/ODS data – the same source of mental health diagnoses as the current study) police-reported family violence was substantially higher than the control sample (*OR* = 3.58 [3.00, 4.28]).

It is noted that the use of public mental health service diagnostic records likely underestimated the number of high-prevalence disorders in this sample – such as substance-use disorders, anxiety, and depression – as these high-prevalence disorders are not routinely treated by the public mental health system, rather by private practitioners (Short et al., 2010). However, the prevalence of other, less common mental health disorders in this sample – such as psychotic disorders – is likely to be a more accurate indication of prevalence given that the majority of individuals with these disorders will have contact with the public mental health system during their lifetime (Wallace et al., 2004).

### The Impact of Family Violence Characteristics on YFV Users’ Psychological Distress and Mental Ill-Health

Contrary to expectations, when accounting for sex and age, a history of FV victimisation was not significantly associated with increased mental health service use or lifetime diagnosis among YFV users. The absence of an association between victimisation and mental health/psychological distress in this sample could potentially be explained by under-representation of victimisation history, given that only police-reported FV victimisation was available. Therefore, it would be remiss to rule out the possibility of an association between these factors, particularly given prior work demonstrating an association between child maltreatment, exposure to FV and YFV (Nowakowski-Sims, 2018; Nowakowski-Sims & Rowe, 2015; Wolfe et al., 2004), as well as mental health (Blum et al., 2019). Furthermore, the victimisation history variable used reflected any FV victimisation, as opposed to FV victimisation in specific developmental periods – which may have different associations with mental health. It would be worthwhile for future studies to utilise multiple data sources that can provide a more complete picture of an individual’s victimisation history (e.g., child protection records).

Although this study found an association between violence use across multiple relationships and mental health outcomes, a causal relationship cannot be assumed. It is equally possible that the circumstances leading the young person to use FV in multiple relationships also contribute to their level of psychological distress, or that the presence of psychological distress and mental health concerns contributes to pervasive difficulties in familial or intimate relationships, and FV. It is also reasonable to hypothesise that a bidirectional relationship may exist. Regardless, this study suggests a relationship between more pervasive use of FV and poorer mental health outcomes, which warrants further, more nuanced investigation.

### Temporal Relationship Between Psychological Distress and YFV Incidents

The findings from this study suggest that young people are more likely to have a mental health service presentation before or around the same time (i.e., within the same 3-month period) as engaging in YFV. There was not enough support for a relationship between YFV use incidents and mental health service presentations in the quarter after. These results largely support our hypothesis.

The limited sample size available for analysis prevents any firm conclusions about the sequencing of mental health concerns and YFV use due to the low granularity of the data. However, the results do indicate some meaningful temporal relationship between mental health service use and YFV use; namely, that young people are more likely to have contact with mental health services prior to engaging in FV behaviours, as opposed to after engaging in FV behaviour. This suggests that psychological distress or mental ill-health could play a role in a young person’s use of FV, and/or that young people typically seek help for psychological distress prior to or close to engaging in violent behaviour towards family members or intimate partners. This extends previous research findings identifying mental health as a correlate of YFV use (Peck et al., 2021) and suggests that further research with a larger sample is required to investigate the time–order relationship between mental health concerns.

It is important to acknowledge that patterns of service use are shaped by accessibility. Young people experiencing significant distress may not always access services in a timely manner, or they may disengage early due to practical or cultural barriers. High dropout rates from mental health services are well documented, with many young people discontinuing treatment before completion (Johnson et al., 2009) – which may partly explain why some young people present to services around the same time as engaging in YFV, but not consistently after. These accessibility challenges may be particularly pronounced for diverse populations, where cultural responsiveness, stigma, or systemic inequities can influence engagement (de Haan et al., 2018). While our findings point to a meaningful temporal link between psychological distress and YFV, further research is needed to better account for how accessibility and engagement with services shape these patterns.

### Limitations

The lack of a suitable comparison group also makes it impossible to draw conclusions about whether the mental health and psychological distress of young people engaged in FV behaviours differ from those of other young people who have not come to the attention of the police for engaging in FV. Ideally, future studies will incorporate a matched comparison group to provide a clearer indication of how mental health and other factors potentially related to YFV differ in this cohort of young people.

Reliance on police-reported YFV incidents is both a limitation and a strength. The sample obviously under-represents the true scope of YFV in the community, given that a minority of YFV cases are reported to police (Fitz-Gibbon et al., 2022). However, the use of administrative data allows for strong data-linkage methodologies that are a useful part of the puzzle when trying to establish incidence and prevalence rates. The findings of the current study are unable to be generalised to all YFV users, but rather are applicable to those young people whose use of violence towards intimate partners and family members has led to contact with the police.

Administrative data is also a limitation and a strength when measuring mental health concerns. The mental health service data allows for a broader conceptualisation of mental health concerns than is possible when restricted to diagnostic categories. If a young person is seeking mental health support, it can be inferred that they or their family feel they have mental health needs, regardless of the diagnosis that is assigned. However, the lack of diagnostic information from the MBS and reasons for presentation to public mental health services are substantial limitations, as it means we were not able to describe the nature of the concerns leading young people to present. That being said, the use of administrative data is a useful approach that can be supplemented by self-report data about mental health, and together these methodologies can be used to build a more comprehensive understanding of the relationship between mental health and FV.

The use of police data to exclusively measure FV victimisation and use in the sample was a further limitation. The use of a broader range of information sources to measure victimisation and YFV use (e.g., child protection files) would have provided a more comprehensive understanding of the extent and nature of YFV. It is essential that future research considers the inclusion of such sources in administrative data-linkage studies to provide a more detailed understanding of the relationship between victimisation, mental health, and YFV.

Police data and administrative mental health data also lacked indicators of diversity within the sample. Without such indicators, we are unable to determine whether there are nuances in mental health presentations across YFV users, which depend on individual differences. The absence of such may impact the potential efficacy of interventions developed based on emerging research (such as the current study). Further research focused on diversity among YFV users is necessary to address this remaining gap in the evidence base.

The generalisability of findings is also constrained by the regional focus on a single police division in Melbourne, Australia. Broader application of the results may be limited by sociocultural and systemic differences across jurisdictions. Furthermore, the sample included young people aged up to 24 years, capturing a wide developmental range. While this aligns with contemporary understandings of extended adolescence, it introduces variability in developmental stages that may influence both YFV behaviours and mental health service presentations. To overcome this, future studies may choose to conduct analyses with the overall sample (young people up to 24 years old) and then by separate age groups, similar to Sheed, McEwan, Simmons et al. (2023).

### Implications for Research and Practice

This study expands the knowledge base regarding mental health among young people using violence against intimate partners and/or family members by establishing the prevalence of psychological distress (as indicated by mental health service use) and associated mental health diagnoses whilst considering the impact of FV characteristics. Furthermore, the investigation of the temporal relationship between mental health and police-reported incidents of YFV has progressed the field closer to establishing a causal relationship between mental health/psychological distress and YFV, albeit with the need for further research with more rigorous methodologies, as highlighted above.

If future research can determine a non-spurious, time–order relationship between psychological distress and YFV, then it strengthens the argument that there is a role for mental health services in the prevention of and response to YFV. However, it is also important to recognise that accessibility issues and high dropout rates may limit the effectiveness of such an approach, as many young people disengage early or are unable to access ongoing care. These challenges highlight the need for services to not only identify YFV risk but also to implement strategies to reduce barriers, improve engagement, and tailor responses to diverse populations. Such responses to YFV would align with the current recommendations that advocate for individualised, non-statutory interventions (Toole-Anstey et al., 2021).

## Conclusion

This study highlights an association between mental health and YFV, demonstrating that psychological distress and mental ill-health are factors in the lives of young people engaging in such behaviours. The findings also provide new evidence that this cohort of young people is more likely to seek help for psychological distress before or around the time that their FV is reported to the police. Future research should endeavour to include matched comparison samples and adopt more rigorous methodologies that allow for causal inference. This will help to build an evidence base that can inform secondary and tertiary prevention strategies for YFV.

## Supplemental Material

sj-docx-1-jiv-10.1177_08862605261444015 – Supplemental material for The Association Between Psychological Distress and Young People Engaging in Family Violence: A Data-Linkage Study Supplemental material, sj-docx-1-jiv-10.1177_08862605261444015 for The Association Between Psychological Distress and Young People Engaging in Family Violence: A Data-Linkage Study by Maddison Riachi, Benjamin Spivak, Troy McEwan and Nina Papalia in Journal of Interpersonal Violence

## Notes

1. Noting that young people may have been listed as Respondents in FV incidents before the sampling period that was used for inclusion in the sample. These incidents prior to the sampling period are captured in the Respondents’ full FV histories.
