# Supplementary material for: The Association Between Psychological Distress and Young People Engaging in Family Violence: A Data-Linkage Study
Source: J Interpers Violence. 2026 May 28;41(19-20):7976–8001. doi: 10.1177/08862605261444015 (PMC13570987; doi:10.1177/08862605261444015)
Supplement: Supplementary file 1 [file sj-docx-1-jiv-10.1177_08862605261444015.docx]

**Supplementary Information**

**Table S1**

*Characteristics of the general population covered by the police division in which family violence incident reports were sampled in the study (Victoria Police North-West Division 4, or ND4), compared with the characteristics of the Victorian general population*

| Characteristic | ND4 Area Population | Victorian Population |
| --- | --- | --- |
|  | *N* = 476,993 | *N* = 5,946,059 |
| Number of family violence incident reports per 100,000 people^a^ | 1,184 | 1,233 |
| Males | 49.1% | 49.0% |
| Females | 50.9% | 51.0% |
| Age |  |  |
| 0-17 years | 22.2% | 21.8% |
| 18-24 years | 9.9% | 9.7% |
| 25-34 years | 17.9% | 15.1% |
| 35-44 years | 14.5% | 13.6% |
| 45+ years | 35.5% | 39.8% |
| Aboriginal and/or Torres Strait Islander people | 0.57% | 0.80% |
| Born in Australia | 59.8% | 64.1% |
| Education |  |  |
| Graduate level or higher | 20.5% | 19.7% |
| Highest level = level 3/4 certificate, advanced diploma, diploma, or school grade 10 or above | 41.2% | 43.2% |
| Highest level = school grade 9 or below | 8.2% | 7.1% |
| Median personal income bracket per annum^b^ | $33,800-$41,599 | $33,800-41,599 |

*Note.* The demographic figures and underlying *N*’s reported in the table were calculated from the 2016 Australian Census (Australian Bureau of Statistics. [2016] 2016 Census - Counting Persons, Place of Enumeration (MB)[Table Builder]. Accessed 14/10/2023). Family violence incident reports were conversely derived from Victorian Police data for the financial year 2017/18, as the same information for 2016 is not publicly available (Crime Statistics Agency. [2022]. Victoria Police Data Tables (2021-22). Accessed 14/10/2023, <https://files.crimestatistics.vic.gov.au/2023-04/Victoria%20Police%20Data%20Tables%20%282021-22%29.xlsx>). The underlying *N*’s to compute a rate of family violence incident reports per 100,000 people for ND4 and Victoria were therefore taken from the 2017 estimated resident population (Australian Bureau of Statistics. [2023]. ABS Estimated resident population 2001-2021 by 2021 LGA [BETA]. Accessed 20/10/2023, <https://digital.atlas.gov.au/datasets/019e1fed4e9f429eaf9189a0a461ead5_1/about>).

^a^ The underlying *N* for ND4 and Victoria were based on estimated resident population size as of 2017 to match the time period of family violence incident reports during this period: Victoria *N* = 6,299,798; ND4 *N* = 528,283;

^b^ The underlying *N* for ND4 and Victoria are based only on those individuals who reported an income in the 2016 census: Victoria *N* = 3,887,629, ND4 *N* = 312,571.

**Table S2**

*Coding Scheme for Mental Health Diagnosis Variable*

| **Variable** | **Data source** | **Variable and Description** | **Description** |
| --- | --- | --- | --- |
| Mental health diagnosis | VAHI | **ICD Code F10-F19 – Mental and Behavioural disorders due to psychoactive substance use**   - F10 (F10.0-F10.9) Mental and behavioural disorders due to use of alcohol - F11 (F11.0-F11.9) Mental and behavioural disorders due to use of opioids - F12 (F12.0-F12.9) Mental and behavioural disorders due to use of cannabinoids - F13 (F13.0-F13.9) Mental and behavioural disorders due to use of sedatives or hypnotics - F14 (F14.0-F14.9) Mental and behavioural disorders due to use of cocaine - F15 (F15.0-F15.9) Mental and behavioural disorders due to use of other stimulants, including caffeine - F16 (F16.0-F16.9) Mental and behavioural disorders due to use of hallucinogens - F17 (F17.0-F17.9) Mental and behavioural disorders due to use of tobacco - F18 (F18.0-F18.9) Mental and behavioural disorders due to use of volatile solvents - F19 (F19.0-F19.9) Mental and behavioural disorders due to multiple drug use and use of other psychoactive substances | Substance-Use Disorders |
|  |  | **ICD Code F20 – F29– Schizophrenia, Schizotypial and delusional disorders**   - F20 (F20.0-F20.9) Schizophrenia - F21 Schizotypal disorder - F22 (F22.0-F22.9) Persistent delusional disorders - F23 (F23.0-F23.9) Acute and transient psychotic disorders - F24 Induced delusional disorder - F25 (F25.0-F25.9) Schizoaffective disorders - F28 Other nonorganic psychotic disorders - F29 Unspecified nonorganic psychosis | Schizophrenia/Psychotic Disorder |
|  |  | **ICD Code F30-F31 – Bipolar or related disorders**   - F30 (F30.0-F30.9) Manic Episode - F31 (F31.0 – F31.9) Bipolar affective disorders   **ICD Code F32-F38 – Depressive Disorders**   - F32 (F32.0-F32.9) Depressive episodes - F33 (F33.0-F.33.9) Recurrent depressive disorders - F34 (F34.0-F34.9) Persistent mood (affective) disorders - F38 (F38.0-F38.8) Other mood (affective) disorders - F39 Unspecified mood (affective) disorder | Mood disorders |
|  |  | **ICD Codes F40-F41 – Anxiety and related disorders**   - F40 (F40.0-F40.9) Phobic Anxiety Disorders - F41 (F41.0-F41.9) Other Anxiety Disorders   **ICD Code F42 – Obsessive compulsive disorders**   - F42 (F42.0-F42.9) Obsessive-compulsive disorders   **ICD Codes (see below) – Childhood anxiety disorders**  - F93.0 – Separation anxiety disorder of childhood  - F93.1 – Phobic anxiety disorder of childhood  - F93.2 – Social anxiety disorder of childhood  **ICD Codes (see below) – Attachment Disorders**  - F94.1 Reactive attachment disorder of childhood  - F94.2 Disinhibited attachment disorder of childhood | Anxiety/Fear/OCD Disorders |
|  |  | **ICD Code F43.1 Post-traumatic stress disorder**   - F43.1 Post-traumatic stress disorder   **ICD Codes F43 – Severe stress and adjustment disorders**   - F43.0 acute stress reaction - F43.2 Adjustment disorder - F43.8 Other rections to severe stress - F43.9 Reaction to severe stress, unspecified | Stress Disorders |
|  |  | **ICD Codes (see below) Any Personality Disorder**  - F60.0 Paranoid Personality Disorder  - F60.1 Schzoid Personality Disorder  - F60.2 Dissocial Personality Disorder  - F60.3 Emotionally Unstable Personality Disorder  - F60.4 Histrionic Personality Disorder  - F60.5 Anankastic Personality Disorder  - F60.6 Anxious Personality Disorder  - F60.7 Dependent Personality Disorder  - F60.8 Other Specific Personality Disorders  - F60.9 Personality Disorder Unspecified  - F61 Mixed personality disorder | Any Personality Disorder |
|  |  | **ICD Codes (see below) Pervasive Developmental Disorders**  - F84.0 Childhood Autism  - F84.1 Atypical Autism  - F84.5 Asperger Syndrome | Neurodevelopmental/Childhood Disorders |
|  |  | **ICD Codes (See below) Hyperkinetic disorders**  - F90 (F90.0-F90.9) Hyperkinetic disorders |  |
|  |  | **ICD Codes (See below) Conduct disorders**  - F91.0 Conduct disorder confined to the family context  - F91.1 Unsocialised conduct disorder  - F91.2 socialised conduct disorder  - F91.8 other conduct disorders  - F91.9 Conduct disorder unspecified  - F92 (F92.0-F92.9) Mixed disorders of conduct and emotions  **ICD Codes (See below) – Oppositional Defiant Disorder**  - F91.3 Oppositional Defiant Disorder |  |
|  |  | **ICD Codes (See below) – Other Childhood and Adolescence Disorders**  - F93.3 Sibling Rivalry Disorder  - F93.8 Other childhood emotional disorders  - F93.9 Childhood emotional disorder, unspecified  - F94.0 Elective mutism  - F94.8 Other childhood disorders of social functioning  - F94.9 Childhood disorder of social functioning, unspecified  - F95 (F95.0-F95.9) Tic Disorders  - F98 (F98.0-F98.9) Other behavioural and emotional disorders with onset usually occurring in childhood and adolescence |  |

**Table S3**

Description of Sample and Outcome Data Sources

| **Source** | **Data extracted** | **Dates of data** | **Purpose** | **Research Question** |
| --- | --- | --- | --- | --- |
| Victoria Police | Involvement in a family violence incident recorded in ND4:   - Sex - Age - Relationship between perpetrator and AFM | September 2016 – June 2017 | Sample | RQ 1 & 2 |
|  | For each individual:   - FV incidents listed as Respondent (either same or different dyad to index incident) - FV incidents listed as AFM (either same or different dyad to index incident) | Lifetime – End January 2018 | Sample – Time-based analysis | RQ3 |
|  |  |  |  |  |
| Medicare Benefits Scheme | - MBS item number - MBS item description - Date of service - The charge by provider - Co-payment (i.e. payment made by the individual) - Medicare benefit amount payable - Indication of whether the procedure was performed in a hospital - Number of services provided | January 2010 – End January 2018 | Outcome – Private Mental Health Service Contact | RQ1, 2, & 3 |
|  |  |  |  |  |
| Victorian Mental Health Data (CMI/ODS) | - CMI/ODS Program Type code - Dates of contacts - Service/s received | January 2010- End January 2018 | Outcome – Public Mental Health Service Contacts | RQ 1, 2, & 3 |
| Victorian Mental Health Data (CMI/ODS) | - Mental health disorder diagnosis/diagnoses | Lifetime until End January 2018 | Outcome – Public mental health diagnoses | RQ 1& 2 |

**Lagged and Lead Analyses**

Lagged variables are when the dependent variable (i.e., mental health service contact) is lagged in time and lead variables are when the dependent variable is shifted ahead in time. The time period specified for the lagged and lead dependent variables for this study was 3 months (one quarter). The lagged mental health service contact variable was created by shifting mental health service contacts 3 months after real time. The lead mental health service contact variable was created by shifting mental health service contacts 3 months ahead of real time. Figure S1 provides a visual representation of the lagging and leading procedure. By producing lagged and lead mental health service contact variables, these can be used to analyse the probability of mental health service contact occurring in any given three-month period (quarter) in relation to the YFV incident. Specifically, in the quarter before, the same quarter, or the quarter immediately following the quarter of interest.

**Figure S1**
Visual Representation of Lagging and Leading Procedure

| **YFV Incidents** | **Real time mental health service contact** | **Lead mental health service contact** | **Lagged mental health service contact** |
| --- | --- | --- | --- |
|  |  | Q1 |  |
| Q1 | Q1 | Q2 |  |
| Q2 | Q2 | Q3 | Q1 |
| Q3 | Q3 | Q4 | Q2 |
| Q4 | Q4 |  | Q3 |
|  |  |  | Q4 |

Note: Q stands for quarter (3 months).

**Table S4**

*Descriptive Statistics for Frequency of Mental Health Service Use for Respondents who accessed Mental Health Services from January 2010 until January 2018 by Service Tier*

|  |  | **YFV Respondents** | **Sex** | | | **Age** | | |  |
| --- | --- | --- | --- | --- | --- | --- | --- | --- | --- |
|  |  |  | | Male | Female | | Under 18 | 18-25 | |
| Tier 1 | *n ^a^* | 2573 | | 1946 | 627 | | 459 | 2114 | |
|  | *Min^b^* | 1 | | 1 | 1 | | 1 | 1 | |
|  | *Q1* | 2.75 | | 2 | 3 | | 1.25 | 3 | |
|  | *Med* | 7 | | 7 | 6 | | 5 | 7.5 | |
|  | *Q3* | 14.25 | | 15 | 13 | | 13.75 | 15 | |
|  | *Max* | 118 | | 118 | 77 | | 46 | 118 | |
|  | *Mean* | 12 | | 12 | 13 | | 9 | 13 | |
|  |  |  | |  |  | |  |  | |
| Tier 2 | *n* | 15309 | | 9666 | 5643 | | 2813 | 12496 | |
|  | *Min* | 1 | | 1 | 1 | | 1 | 1 | |
|  | *Q1* | 14.5 | | 11 | 30 | | 29.5 | 13.75 | |
|  | *Med* | 61 | | 61 | 61.5 | | 114 | 59.5 | |
|  | *Q3* | 166.5 | | 153 | 197 | | 179.5 | 153.25 | |
|  | *Max* | 2232 | | 2232 | 1413 | | 389 | 2232 | |
|  | *Mean* | 133 | | 119 | 166 | | 122 | 136 | |
|  |  |  | |  |  | |  |  | |
| Tier 3 | *N* | 3161 | | 1548 | 1613 | | 890 | 2271 | |
|  | *Min* | 1 | | 1 | 1 | | 1 | 1 | |
|  | *Q1* | 4.5 | | 4 | 9 | | 8.5 | 4 | |
|  | *Med* | 13.5 | | 11 | 25 | | 15 | 12 | |
|  | *Q3* | 29.25 | | 25.75 | 56.5 | | 23 | 31 | |
|  | *Max* | 659 | | 321 | 659 | | 659 | 321 | |
|  | *Mean* | 34 | | 23 | 58 | | 59 | 29 | |
|  |  |  | |  |  | |  |  | |

Note: ^a^ *n* represents total number of mental health service contacts/admissions within service tier across all YFV users, ^b^ the five-point descriptive data summary is of YFV users with mental health service contacts/admissions within the respective categories. Tier 1 = private community mental health service contacts, Tier 2 = public community mental health service contacts, and Tier 3 = inpatient and acute public mental health service contacts.
